# Enhanced skin adhesive property of electrospun α-cyclodextrin/nonanyl group-modified poly(vinyl alcohol) inclusion complex fiber sheet†

**DOI:** 10.1039/d1ra00422k

**Published:** 2021-02-25

**Authors:** Xi Chen, Tetsushi Taguchi

**Affiliations:** Polymers and Biomaterials Field, Research Center for Functional Materials, National Institute for Materials Science 1-1 Namiki, Tsukuba Ibaraki 305-0044 Japan taguchi.tetsushi@nims.go.jp

## Abstract

Many medical tapes on the market lack sufficient adhesive strength and breathability. Owing to its high biocompatibility, poly(vinyl alcohol) (PVA), a synthetic polymer, has attracted attention in the medical field. In this study, we aimed to prepare an inclusion complex fiber (ICFiber) using α-cyclodextrin (α-CD) and nonanyl-group-modified PVA (C9–PVA) for skin adhesion with improved performance. By changing the concentration of α-CD, six microfiber sheets were fabricated by electrospinning the α-CD/2.3C9–PVA inclusion complex solutions. The bonding strength and energy of the ICFiber sheets on the porcine skin were evaluated. Among the tested ICFiber sheets, the ICFiber-3 (molar ratio of α-CD/C9 groups was 0.612) sheet showed high tensile strength and break strain. The bonding strength and energy of ICFiber-3 sheet on porcine skin were 1.10 ± 0.11 N and 5.07 ± 0.94 J m^−2^, respectively, in the presence of water. In addition, ICFiber-3 sheet showed a better water vapor transmission rate (0.95 ± 0.02 mL per day) than commercial tapes. These results demonstrate that the α-CD/2.3C9–PVA ICFiber sheet is a promising adhesive for wearable medical devices.

## Abstract 2

Inclusion complex fiber (ICFiber) sheets composed of α-cyclodextrin (α-CD) and nonanyl-group-modified poly(vinyl alcohol) (PVA) (C9–PVA) were developed for breathable skin adhesive.

## Graphical abstract

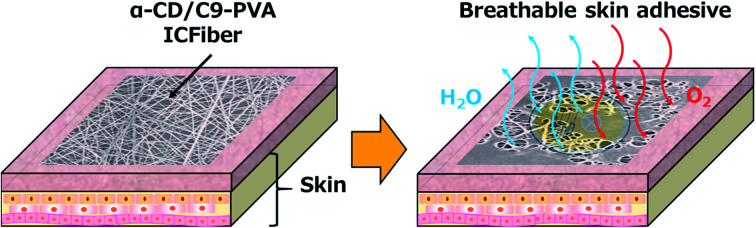

## 1 Introduction

Recently, monitoring human health using wearable sensors has received a great deal of attention.^1^ The wearable sensor should be in close contact with the skin to detect small signals from the human body. However, the human skin is relatively rough and has a microstructure called the skin pattern, which prevents the detector from adhering to it firmly. To avoid detachment, wearable sensors are usually attached to the skin *via* an adhesive medical tape or a wound dressing.^2^ Although medical tapes can be strongly attached to the skin, they detach upon sweating; thus, affecting the performance of the sensor.^3^ In addition, the non-breathable adhesive traps moisture under the tape, which can damage the skin due to prolonged wetting. Furthermore, continued exposure to some acryl-based adhesives may cause inflammation or allergic reactions.^4^ Recently, poly(dimethylsiloxane) (PDMS)-based wearable sensors that can be directly applied to the skin without a fixing tape have been developed.^5,6^ These devices possess characteristic patterns that help them adhere strongly to the skin in both dry and wet environments. However, complex manufacturing processes have hindered the practical application of these sensors. Poly(vinyl alcohol) (PVA) is a versatile hydrophilic polymer, which has been widely used in biotechnology and biomedicine owing to its excellent physicochemical properties and high biocompatibility.^7^ Currently, PVA is being utilized to develop wearable sensors owing to its good adhesiveness and biocompatibility^8,9^ under dry conditions. However, hydrophilic PVA detaches easily from the skin after sweating, negatively influencing the performance of the sensor.

In our previous studies, alkyl group-modified biopolymers were designed to prepare surgical sealants,^10–12^ porous membranes,^13^ and microparticles^14,15^ for adhesion to soft biological tissues. We observed that hydrophobic modifications can improve the soft tissue adhesion of hydrophilic polymers under wet conditions. The key driving forces that increase the interfacial strength are the anchoring effect of the hydrophobic groups on phospholipid membranes, and enhancement of the interactions between the amphiphilic polymers and extracellular proteins.^12,16^ Based on these findings, we prepared hydrophobically modified PVA (hm-PVA) films and evaluated their adhesion to porcine skin under wet conditions.^17^ The resulting films showed good adhesion properties on porcine skin; however, their adhesion was not stable after exposure to moisture. One strategy for improving adhesion stability involves the use of hm-PVAs with long alkyl groups. However, the long alkyl groups can easily aggregate, which renders the anchoring to the phospholipid membranes of the corneocytes in the stratum corneum, and the interaction with the keratinocyte lipids (ceramide, fatty acid, and cholesterol) difficult. Therefore, we focused on adding α-cyclodextrin (α-CD), which possesses temperature-responsive inclusion/de-inclusion properties.^18^ The solubility of α-CD in water (145 mg mL^−1^ at 25 °C) is high and the inner diameter of the hydrophobic pocket (4.7 Å) is suitable for inclusion of alkyl chain. We hypothesized that α-CD can temporarily prevent the aggregation of alkyl groups and improve adhesion to the skin. In addition, a porous structure can be easily produced by electrospinning, and the gas permeability is expected to improve.

**Scheme 1:**
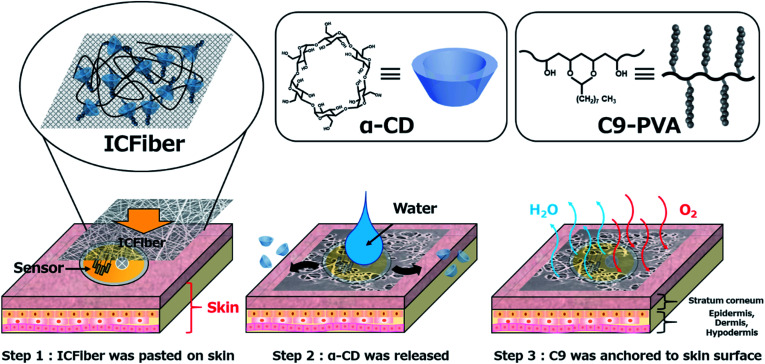
Schematic illustration of the adhesion mechanism of inclusion complex fiber (ICFiber) to skin. The ICFiber forms a stable C9–PVA layer on the surface of the skin due to release of α-CD in an aqueous environment. After releasing α-CD, the C9–PVA layer can adhere well to skin by anchoring to the stratum corneum.

Herein, we aimed to develop an inclusion complex fiber (ICFiber) consisting of α-CD and nonanyl-group-modified PVA (C9–PVA) for skin adhesion (Scheme 1). Relying on the inclusion ability of α-CD for the alkyl groups and the water insolubility of C9–PVA, microfiber sheets were fabricated by electrospinning the α-CD/2.3C9–PVA inclusion complex solutions. Furthermore, skin adhesion on porcine skin and water vapor permeability of ICFiber sheets were evaluated.

## 2 Experimental

### 2.1. Materials

Ethanol (EtOH, 99.5%), dimethyl sulfoxide (DMSO), 6 N hydrochloric acid (HCl), 10% formalin neutral buffer solution, α-cyclodextrin (α-CD), and 99.9% dimethyl sulfoxide-d_6_ (DMSO-d_6_) containing 0.05% w/v tetramethysilane (TMS) were purchased from Wako Pure Chemical Industries, Ltd. (Osaka, Japan). Otsuka physiological saline 2-port was purchased from Otsuka Pharmaceutical Co., Ltd. (Tokyo, Japan). PVA (*M*_w_ = 88 000, saponification degree > 98.5%) was purchased from Nacalai Tesque, Inc. (Kyoto, Japan). Nonanal (C9) was purchased from Tokyo Chemical Industry Co., Ltd. (Tokyo, Japan). Super glue LPJ-005 was purchased from Henkel Co. (Tokyo, Japan). 3 M scotch tape and Tegaderm™ film were purchased from 3 M Co., Ltd. (Saint Paul, USA). Elastikon® elastic tape was purchased from Johnson & Johnson Co., Ltd. (New Brunswick, NJ, USA). The porcine skin was washed with PBS three times and shaved with a clipper before the experiment (Thrive model 515R, Daito Electric Industry, Osaka, Japan). Porcine skin was used within 48 h of slaughter.

### 2.2. Synthesis of C9–PVAs

C9–PVAs were synthesized *via* the nucleophilic substitution reaction between an aldehyde and hydroxyl groups of PVA, as reported previously.^17,19–22^ Briefly, a 5% w/v solution of PVA was prepared in a mixed solution (H_2_O/DMSO = 1 : 3 (v/v), 0.01 M HCl), and different amounts of aldehyde groups (nonanal, C9) were added to the PVA solution. The resulting mixture was stirred at 50 °C for 1 h. A reflux condenser was used in all processes. After the reaction, the obtained C9–PVA solution was added to 600 mL of cold EtOH while stirring for 1 h. Unreacted aldehyde and DMSO were removed by washing with EtOH. Finally, the precipitated C9–PVA was evaporated under vacuum as a white crystal. All crystals were finely crushed using a crusher for further experiments (Wonder Crusher WC-3, Osaka Chemical, Osaka, Japan).

### 2.3. Characterization of C9–PVA

The chemical structures of the C9–PVAs were determined using a proton nuclear magnetic resonance (^1^H-NMR) spectroscopy (JNM-ECS-400, JEOL, Tokyo, Japan) and a 0.5% w/v C9–PVA/DMSO-d_6_ solution at 25 °C. Eight scans were performed for each sample. Fourier transform-infrared (FT-IR) spectroscopy (8400S, Shimadzu, Kyoto, Japan) analysis was performed to confirm the presence of alkyl groups in the C9–PVA. The scan range was from 700 cm^−1^ to 4000 cm^−1^, with each sample being scanned 64 times. The hydrophobic group modification ratios of the C9–PVAs were calculated using ^1^H-NMR spectroscopy and the following equation:Modification ratio (mol%) = [{ integral area (–CH_3_ proton)/3}/integral area (–CH– proton)] × 100.where integral area (–CH– proton) is the area of the peak at 3.08 ppm corresponding to the αCH proton in the backbone of PVA and C9–PVAs, and integral area (–CH_3_ proton) is the area of the peak at 0.75 ppm assigned to the CH_3_ proton in the hydrophobic groups of C9–PVAs.

### 2.4. Fabrication and characterizations of PVA fiber and ICFiber sheets

Microfiber sheets were fabricated by electrospinning α-CD/C9–PVA inclusion complex solutions. Briefly, PVA and 2.3C9–PVA were dissolved in a 50% ethanol aqueous solution at a concentration of 50 mg mL^−1^ by heating in an autoclave (LSX-500, TOMY) at 90 °C for 10 min. Then, different amounts of α-CD (0–25 mM) were added to a C9–PVA 50% ethanol aqueous solution to form an α-CD/2.3C9–PVA inclusion complex solution. Electrospinning was conducted using a NANON-03 instrument (MECC, Japan) at room temperature (25 °C) (Fig. 2a). PVA and α-CD/C9–PVA inclusion complex solutions were extruded from a 22 G needle at a feed rate of 3 mL h^−1^ at a voltage of 20 kV. The obtained PVA fiber and ICFiber sheets were observed using a scanning electron microscope (SEM) (S-4800 ultrahigh-resolution SEM, HITACHI, Japan).

### 2.5. Determining the tensile strength and break strain of PVA fiber and ICFiber sheets

The tensile strengths of the PVA fiber and ICFiber sheets were determined according to ASTM D638 and measured using a texture analyzer (TA-XT2i; Stable Micro Systems, Godalming, UK) at a tracking speed of 5 mm min^−1^.

### 2.6. Measuring the surface wettability of PVA fiber and ICFiber sheets

To evaluate the hydrophobicity of the fiber sheets, water contact angle (WCA) measurements were conducted using a DropMaster DM-700 (Kyowa Interface Science, Japan). Contact angle measurement was initiated 1 s after a 2 μL deionized (DI) water droplet was dropped on the fiber sheet and continued for up to 15 min. The surface contact angle of the DI water droplet on the fiber sheet was analyzed using the FAMAS software.

### 2.7. Measuring the bonding strength of PVA fiber and ICFiber sheets

The bonding strength of the fiber sheets attached to the porcine skin was determined according to ASTM F2258-05. Briefly, porcine skin was cut into 25 mm × 25 mm squares and placed on the surface of a heater set to 37 °C. A sterile cotton surgical gauze was used to remove excess moisture from the skin surface. Fiber sheets (25 mm × 25 mm) were applied to the skin surface and sprayed with 40 μL of DI water. The bonding force and energy of the fiber sheets were measured using a texture analyzer (TA-XT2i; Stable Micro Systems, Godalming, UK) at an applied force of 2 N, waiting time of 3 min, and tracking speed of 10 mm min^−1^. To elucidate the adhesion mechanism, a cross-section of the porcine skin was fixed in 10% formalin neutral buffer solution, stained with hematoxylin and eosin (H&E), and then observed under an inverted fluorescence phase contrast microscope (BZ-X700; Keyence Co., Tokyo, Japan).

### 2.8. Determining the water vapor transmission rate of PVA fiber and ICFiber sheets

The water vapor transmission rate (WVTR) of each film was measured using a modified wet cup method, according to ASTM E 96 Procedure B. Glass bottles with a 3.14 cm^2^ transmission area were used. Three measurements were performed to calculate water vapor permeability. First, the glass bottle containing DI water (20 mL) was sealed using fiber sheets and then placed in an incubator to maintain the temperature at 37 °C. Silica gel was used to maintain the relative humidity in the incubator. The amount of water lost from the glass bottles was monitored as a function of time. WVTR was calculated using the following equation:WVTR = *V*/*T*where *V* is the volume change of DI water and *T* is the incubation time.

### 2.9. Statistical analysis

Statistical analysis was performed using Tukey's multiple comparisons test of one-way analysis of variance (ANOVA) using GraphPad Prism software. *P* < 0.05 was considered statistically significant. Data are presented as mean ± standard deviation (SD).

## 3 Results and discussion

### 3.1. Synthesis and characterization of C9–PVA

**Fig. 1:**
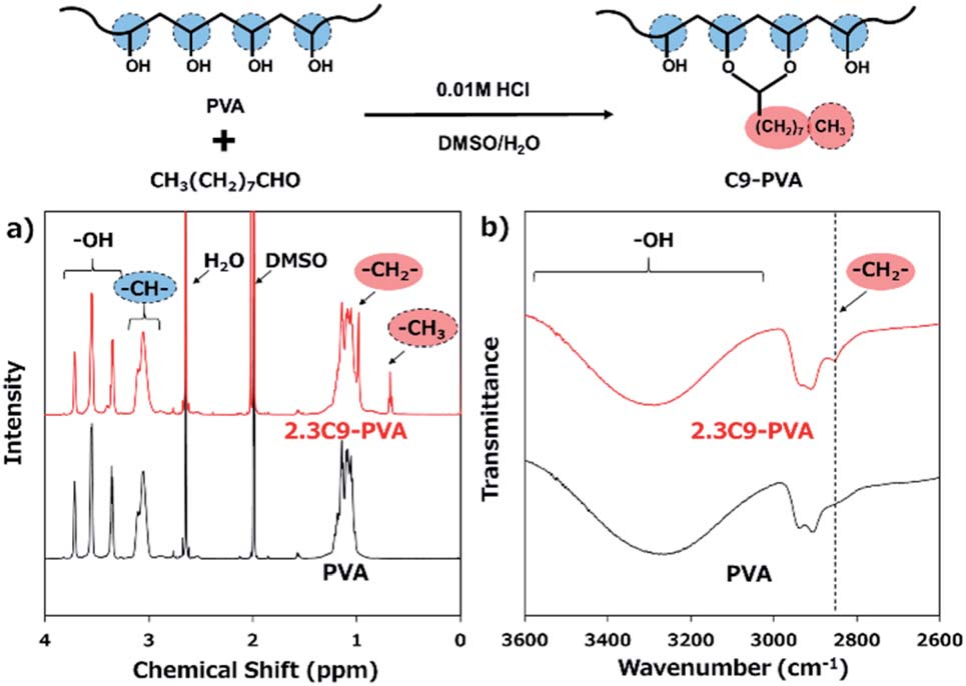
Synthesis and characterization of C9–PVA. (a) ^1^H-NMR spectrum of the C9–PVA/DMSO-d6 solution. (b) FT-IR spectra of the C9–PVA.

The C9–PVAs were prepared *via* the reaction between the hydroxyl groups of PVA and nonanal (Fig. 1).^17,19–22^ Under acidic conditions, the aldehydes quicky react with the hydroxyl groups *via* nucleophilic substitution to form a stable hexagonal ring structure. Fig. 1a shows the ^1^H-NMR spectra of the C9–PVAs with different modification ratios. The typical chemical shifts at 0.68 ppm and 0.98 ppm were assigned to the CH_3_ and αCH_2_ protons, respectively, in the alkyl groups of C9–PVAs, which indicated the successful introduction of the nonanyl groups into the PVA backbone. The modification ratio of the nonanyl groups (2.3 mol%) was calculated from the ^1^H-NMR results. Furthermore, C9–PVA was analyzed using FT-IR spectra (Fig. 1b). The peak observed at 2850 cm^−1^ was attributed to the C–H stretching vibrations of –CH_2_– in the alkyl groups of C9–PVAs. Taken together, these results demonstrate successful C9–PVA synthesis.

### 3.2. Fabrication and characterizations of PVA fiber and ICFiber sheets

**Fig. 2:**
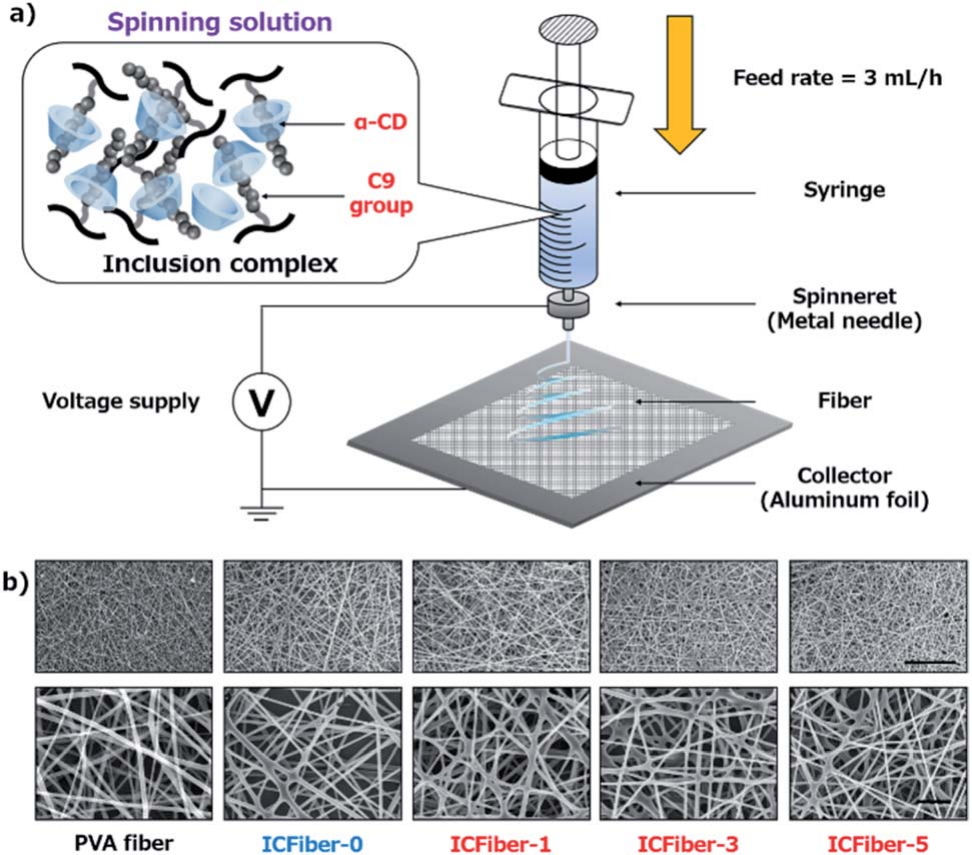
Fabrication and characterization of electrospun fiber sheets. (a) Electrospinning of PVA and α-CD/C9–PVA inclusion complexes into fiber sheets. (b) SEM images of PVA fiber and ICFiber sheets (scale bar of figures above = 50 μm, scale bar of figures below = 10 μm).

Next, we attempted to confirm the formation of the α-CD/C9 group inclusion complex, as shown in Fig. S1a.† It is well known that α-CD can include alkyl units in the aqueous environment to form a white suspension.^23^ Previous reports indicate that alkyl chain (C10) can be included in the hydrophobic pocket of two α-CDs.^24^ Thus, theoretically two α-CDs would be needed to completely include nonanal (C9 group). The results shown in Fig. S1b† confirmed the formation of the α-CD/nonanal inclusion complex. Therefore, it is considered that a stable α-CD/2.3C9–PVA inclusion complex was also formed in an aqueous environment. Microfiber sheets were fabricated by electrospinning (Fig. 2a). At 25 °C, the original PVA solution or the α-CD/2.3C9–PVA inclusion complex solutions (compositions shown in Table 1) were extruded using a 22 G needle at a feed rate of 3 mL h^−1^ at voltages of 15–30 kV. To obtain well-structured fiber sheets, optimization was performed using a 40–60% ethanol aqueous solution (Fig. S2†).

**Table 1:** Components of fiber-making solution

Abbreviation	α-CD (mM)	PVA or 2.3C9–PVA (mg mL^−1^)	Molar ratio (α-CD/C9 groups)
PVA fiber	0	50	—
ICFiber-0	0	50	—
ICFiber-1	5	50	0.204
ICFiber-2	10	50	0.408
ICFiber-3	15	50	0.612
ICFiber-4	20	50	0.816
ICFiber-5	25	50	1.020

A 40% ethanol aqueous solution did not volatilize during electrospinning; thus, the fiber sheets dissolved. In contrast, the 50 or 60% ethanol aqueous solution yielded uniform fiber sheets. However, as a result of the low solubility of α-CD in the 60% ethanol aqueous solution, the 50% ethanol aqueous solution was preferred. In addition, the effect of the voltage applied during electrospinning was also studied. It was noted that electrospun fiber sheets using different α-CD/2.3C9–PVA solutions (5 w/v % 2.3C9–PVA, 0–25 mM α-CD) at a voltage of 20 kV were relatively better than those under other voltages (Fig. S3†). Under these optimized conditions, the fiber sheets produced at different ratios of α-CD had good structures with similar diameters (Fig. 2b).

### 3.3. Determining the tensile strength and break strain of PVA fiber and ICFiber sheets

**Fig. 3:**
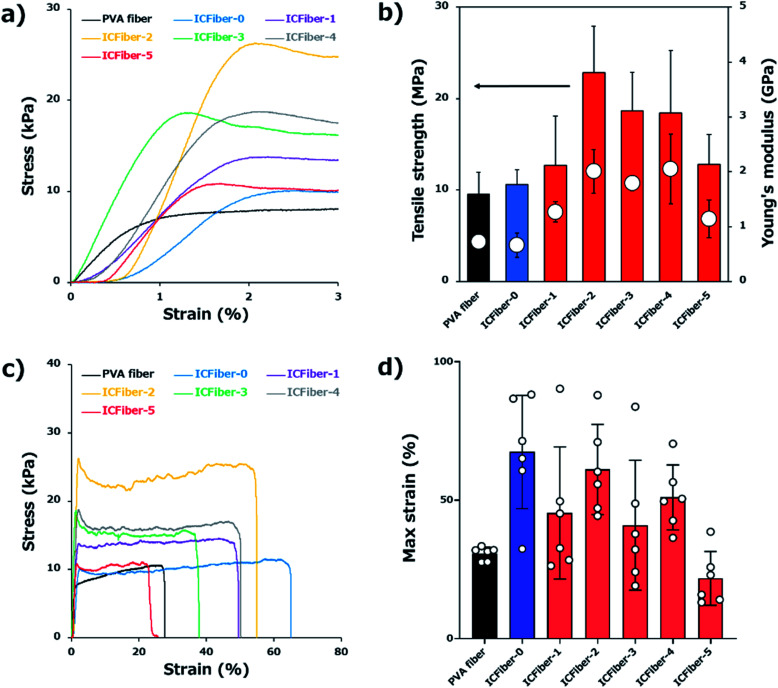
(a) Tensile stress–strain diagram (partial view, 0–3% strain) of PVA fiber and ICFiber sheets. (b) The tensile strength and Young's modulus of PVA fiber and ICFiber sheets. (c) Tensile stress–strain diagram (overall view) of PVA fiber and ICFiber sheets. (d) The break strain of PVA fiber and ICFiber sheets. Data are presented as mean values ± S.D.

Since flexibility of fiber sheets is required when they are applied to the skin, their tensile strength was measured. The stress–strain curves (0–3% strain, Fig. 3a), and the tensile strength and Young's modulus (Fig. 3b) of the PVA fiber and ICFiber sheets are shown. The tensile strength of the PVA fiber and ICFiber-0 (2.3C9–PVA fiber) was approximately 10 MPa. An increase in the tensile strength of the ICFiber sheets (10.6 ± 1.6–22.8 ± 5.0 MPa) was observed with an increasing amount of α-CD of up to 10 mM (ICFiber-2) because of the enhanced intermolecular hydrophobic interaction. Further addition of α-CD did not yield a significant increase in tensile strength of the ICFiber-3 and ICFiber-4 sheets, while that of ICFiber-5 (12.8 ± 3.3 MPa) dramatically reduced. This occurs because at 25 mM of α-CDs, the molar ratio of α-CDs to the C9 groups exceeds 1 : 1; thus, the hydrophobic interaction between the C9–PVA molecules decreases by complete inclusion. The same tendency was observed for the Young's modulus of the ICFiber sheets. The Young's modulus of the PVA fiber and ICFiber-0 sheets was approximately 0.7 GPa, while that of the ICFiber-2 sheet increased to 2.0 GPa ± 0.4 MPa. Further, the decrease in the Young's modulus of ICFiber-5 can be attributed to the presence of excess α-CD.

Fig. 3c shows the stress–strain curves (overall) of the PVA fiber and ICFiber sheets. Interestingly, the break strain of the ICFiber-0 sheet (67.4 ± 20.5%) was twice that of the PVA fiber sheet (30.7 ± 20.5%, Fig. 3d). Due to the absence of α-CD in ICFiber-0, the C9 groups aggregated resulting in weak intermolecular interactions; thus, the ICFiber-0 sheet was easier to stretch. However, the hydrophobic interactions between C9 groups worked with the addition of α-CD. As shown in Fig. 3b and c, the mechanical strength of ICFiber-1–ICFiber-4 increased compared to that of ICFiber-0, but they were difficult to stretch. At 25 mM α-CD (molar ratio > 1 : 1), the C9 groups were completely encapsulated resulting in the lower elasticity of ICFiber-5 compared to that of PVA.

### 3.4. Surface wettability of PVA fiber and ICFiber sheets

**Fig. 4:**
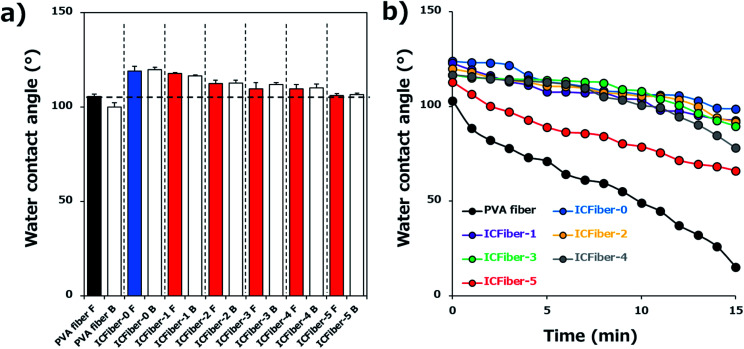
Surface contact angle measurements. (a) WCA of both sides of PVA fiber and ICFiber sheets right after dropping droplets (*n* = 5). (b) Quantification of the decrease in contact angle over time.

Surface wettability may affect the water absorption rate; therefore, it was evaluated by measuring the WCA of each fiber sheet. Fig. 4a shows the behavior of the DI water droplets on the PVA fiber and ICFiber sheets on both sides (front and back). There was no obvious difference in water wettability between the front and back surfaces of the fiber sheets, as hydrophobic units gathered on both the surfaces of the fiber during the electrospinning process. One second after dropping the DI water droplets, the WCAs of the ICFiber sheets were already larger than that of the PVA fiber (105.5° ± 1.4°). Among them, ICFiber-0 had the highest WCA (119.0° ± 2.5°), indicating that the alkyl chains successfully integrated onto the surface of the C9–PVA. In addition, the WCA of the ICFiber sheets decreased as the amount of α-CD (106.1 ± 0.8° for ICFiber-5) increased. When α-CD was added, the remaining alkyl chains were included in the hydrophobic pocket of α-CD; thus, a negligible change in water wettability was observed. Fig. 4b shows the changes in the WCA of the DI water droplets on the fiber sheets over a period of 15 min, and all fiber sheets exhibit a decrease. However, the decrease in WCA (103° to 15.2°) of PVA fiber over time was larger than that of the others because of its high water solubility. The 2.3C9–PVA was water-insoluble, and ICFiber-0 maintained a high WCA (98.6°) even after 15 min. As expected, the addition of α-CD improved the water solubility of 2.3C9–PVA and reduced the WCA of ICFiber sheets over time.

### 3.5. Adhesion test of PVA fiber and ICFiber sheets

**Fig. 5:**
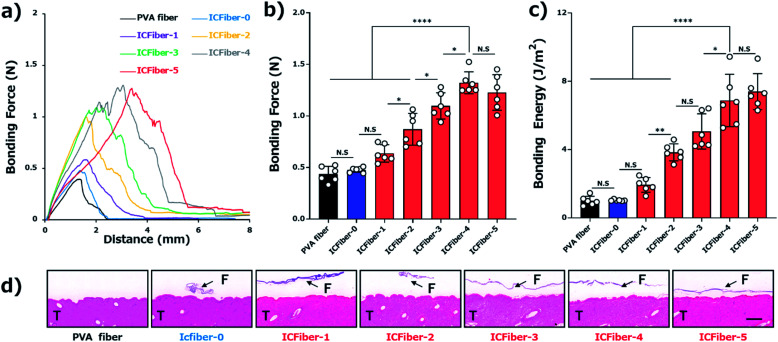
Evaluation of adhesive strength of fiber sheets. (a) Force–distance curve for measuring bonding strength on porcine skin sprayed with DI water. (b) Bonding force of PVA fiber and ICFiber sheets on porcine skin. (c) Bonding energy of PVA fiber and ICFiber sheets on porcine skin. Data are presented as mean values ± SD (*P < 0.05, **P < 0.01, ****P < 0.0001, *n* = 6). (d) Histology of film-porcine skin interface after bonding strength measurement (F = fiber; T = tissue; scale bar: 2 mm).

The adhesive properties of the fiber sheets were investigated by measuring their bonding strengths. According to ASTM F2258-05, the force–distance curve was recorded (Fig. 5a), and the curve peak, area under the curve, bonding force (Fig. 5b) and energy (Fig. 5c) of the fiber sheets on porcine skin were obtained. Fig. 5b clearly shows that the bonding force of the PVA fiber sheet (0.44 ± 0.07 N) was close to that of ICFiber-0 (0.48 ± 0.02 N), as most of the introduced C9 groups would aggregate in the absence of α-CD. The presence of α-CD is expected to inhibit the aggregation of the C9 groups. It was observed in the bonding force of the ICFiber-1–ICFiber-4 sheets, which increased with an increasing amount of α-CD.

The stratum corneum on the surface of the skin is mainly composed of keratinocytes and lipids (the main components are ceramide, free cholesterol, and free fatty acids).^25^ The alkyl groups (C9) introduced into the C9–PVA have similar alkyl chains to those in the lipids. The C9 group is thought to anchor to the lipid bilayer membrane of the keratinocytes or to have a hydrophobic interaction with the lipids. As a result, the ICFiber sheets adhere strongly to the skin surface. The bonding force of ICFiber-4 (1.32 ± 0.10 N) and ICFiber-5 (1.23 ± 0.16 N) sheets was almost three-fold higher than that of the PVA fiber sheet. This may be because ICFiber-4 can cover and penetrate the skin surface efficiently, allowing the C9 groups to easily anchor to the lipid bilayer membrane and exert high interfacial adhesion. The bonding energy of the fiber sheets (Fig. 5c) has the same tendency as the bonding force. The bonding energy of ICFiber-5 (7.40 ± 0.16 J m^−2^) was seven-fold higher than that of the PVA fiber (0.99 ± 0.25 J m^−2^) and ICFiber-0 (1.03 ± 0.04 J m^−2^) sheets. Fig. 5d shows the H&E-stained sections of the porcine skin with the respective fiber sheets (PVA and ICFiber-0–ICFiber-5) after the bonding strength measurement. The PVA fiber sheet completely disappeared as it dissolved in water, whereas the ICFiber sheets remained on the tissue surface. It was observed that ICFiber-0 aggregated on the porcine surface owing to the strong hydrophobic interactions of C9. As the amount of α-CD added increased, the aggregation of the C9 groups was suppressed and the adhesion of the fiber sheets to the porcine skin surface improved.

### 3.6. WVTR of α-CD/C9–PVA ICFiber sheets

**Fig. 6:**
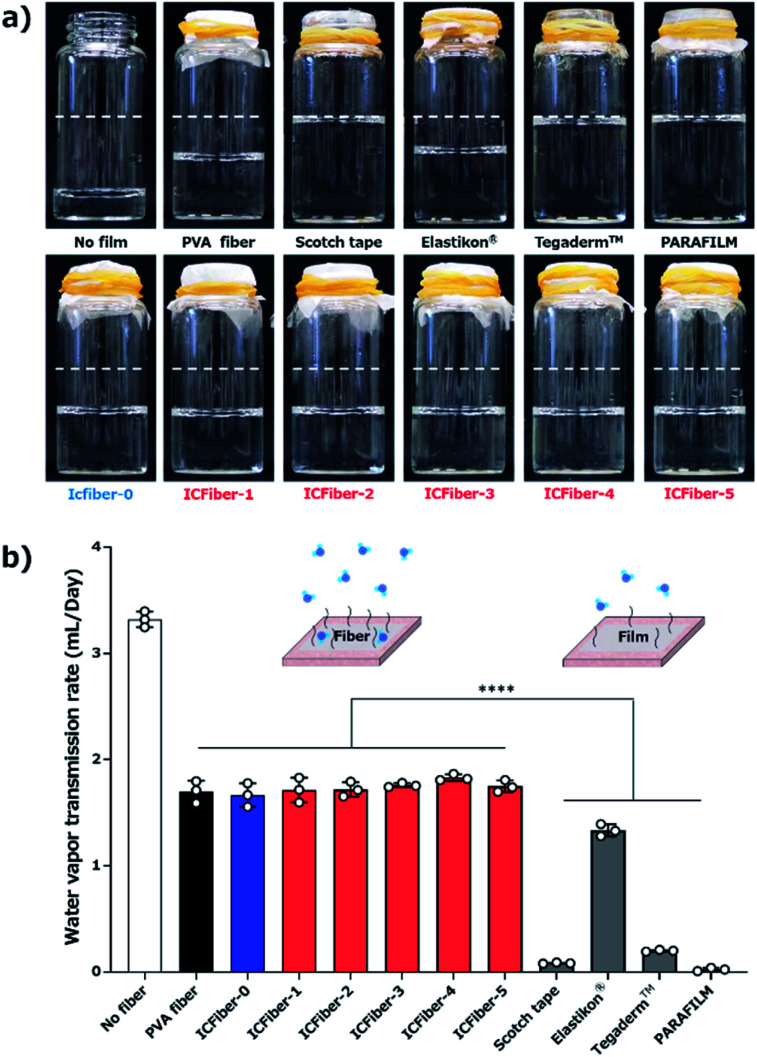
Measurement of water vapor transmission rate (WVTR). (a) Photograph showing the amount of residual water after 7 days of incubation. (b) WVTR of PVA fiber and ICFiber sheets compared to those of commercial products (3 M scotch tape, Elastikon® elastic tape, Tegaderm™ film and PARAFILM). Data are presented as mean values ± SD (****P < 0.0001, *n* = 3).

Prolonged adherence to the skin may cause the fiber sheets to become damp; therefore, determining their water vapor permeability is crucial. The WVTR of each film was measured according to ASTM E 96 Procedure B. Fig. 6a shows the amount of residual water after seven days of incubation at 37 °C, and the results are summarized in Fig. 6b.

**Fig. 7:**
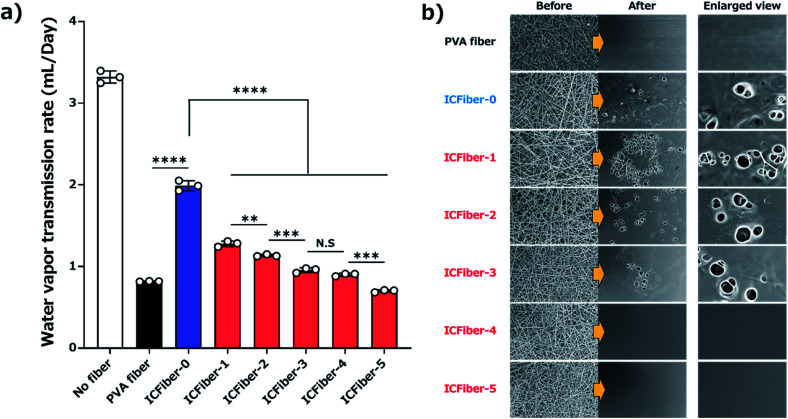
Measurement of water vapor transmission rate (WVTR). (a) WVTR of PVA fiber and ICFiber sheets after spraying DI water. Data are presented as mean values ± SD (**P < 0.01, ***P < 0.001, ****P < 0.0001, *n* = 3) (b) SEM observation of PVA fiber and ICFiber sheets before and after spraying with DI water.

The WVTRs of the PVA fiber and ICFiber sheets without spraying DI water (approximately 1.7 mL per day) were higher than those of commercially available tapes because of their highly porous structure. The 3 M scotch tape has a non-breathable support layer, which results in a limited transmission of water. A similar phenomenon was observed in the moisture-proof thin parafilm. The commercial Elastikon® elastic tape and Tegaderm™ film have pores that allow the skin to breathe; hence, it is possible to avoid skin stiffness during prolonged application. It was observed that the WVTRs of Elastikon® elastic tape and Tegaderm™ film were 1.33 ± 0.05 mL per day and 0.19 ± 0.01 mL per day, respectively. The values were lower than that of the ICFiber sheets, which may have a higher density of pores. The PVA fiber and ICFiber sheets were applied to the skin surface by spraying DI water, and their WVTRs were measured (Fig. 7). It was noted mL per day because the original PVA is highly water soluble. The WVTRs of the ICFiber sheets decreased with an increase in the amount of α-CD, owing to the increase in water solubility. The PVA fiber, ICFiber-4, and ICFiber-5 sheets were completely dissolved, changing the structure of the film (Fig. 7b). However, that the WVTR of the PVA fiber sheet decreased to 0.82 ± 0.01 even though the fiber structure of the ICFiber sheets was lost, the WVTRs of the ICFiber sheets were higher than those of the commercial Tegaderm™ film owing to their hydrophilicity, and water eventually evaporated from the film surface. Given its intact structure and good WVTR, ICFiber-3 can adhere well to the skin surface and prevent stiffness.

## 4 Conclusions

In this study, ICFiber sheets with skin adhesive properties were fabricated with an inclusion complex formation between the nonanyl group-modified PVA and α-CD. It was noted that through electrospinning at 20 kV with a 50% ethanol aqueous solution, the inclusion complex with different concentrations of α-CD (ICFiber-0–5) can form uniform fiber structures. In particular, the ICFiber-3 sheet showed the high tensile strength, break strain, and bonding strength (1.10 ± 0.11 N) and energy (5.07 ± 0.94 J m^−2^). In the presence of water, the ICFiber-3 sheet adhered well to the skin, and also demonstrated better WVTR (0.95 ± 0.02 mL per day) than commercial tapes, which allows the skin to breath under prolonged exposure. Therefore, these results demonstrate that the ICFiber-3 sheet can potentially be used as a base material for wearable medical devices.

## Data availability

The raw and processed data required to reproduce these findings cannot be shared at this time due to technical limitations.

## Conflicts of interest

There are no conflicts to declare.

## Supplementary Material

RA-011-D1RA00422K-s001
