# Supplementary material for: Enhanced skin adhesive property of electrospun α-cyclodextrin/nonanyl group-modified poly(vinyl alcohol) inclusion complex fiber sheet
Source: RSC Adv. 2021 Feb 25;11(15):8759–66. doi: 10.1039/d1ra00422k (PMC8695218; doi:10.1039/d1ra00422k)
Supplement: RA-011-D1RA00422K-s001 [file RA-011-D1RA00422K-s001.pdf]

Electronic Supplementary Material (ESI) for RSC Advances.
This journal is © The Royal Society of Chemistry 2021

Supporting Information

Enhanced Skin Adhesive Property of Electrospun α-cyclodextrin/Nonanyl Group-modified Poly(vinyl alcohol) Inclusion Complex Fiber Sheet

Xi Chen and Tetsushi Taguchi *

Polymers and Biomaterials Field, Research Center for Functional Materials, National Institute for Materials Science, 1-1 Namiki, Tsukuba, Ibaraki 305-0044, Japan

First author

Tel: +81-29-851-8746

Fax: +81-29-860-4752

E-mail: CHEN.Xi2@nims.go.jp

*Corresponding author

Tel: +81-29-851-4498

Fax: +81-29-860-4752

E-mail: TAGUCHI.Tetsushi@nims.go.jp

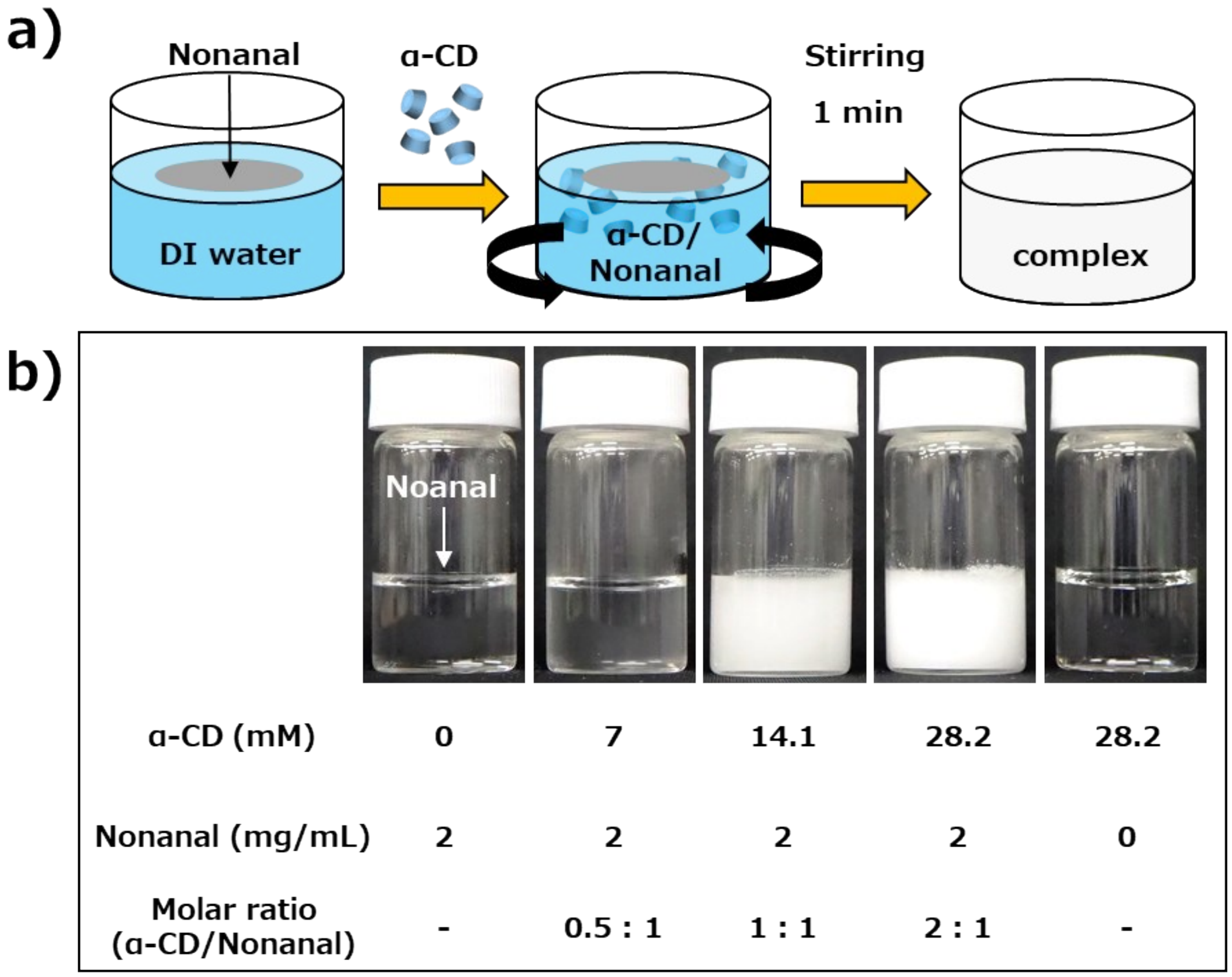

Figure S1. Confirmation of inclusion complex formation. a) The procedure for preparing α-CD/nonanal inclusion complex. b) Photographs showing α-CD/nonanal inclusion complex formation in DI water.

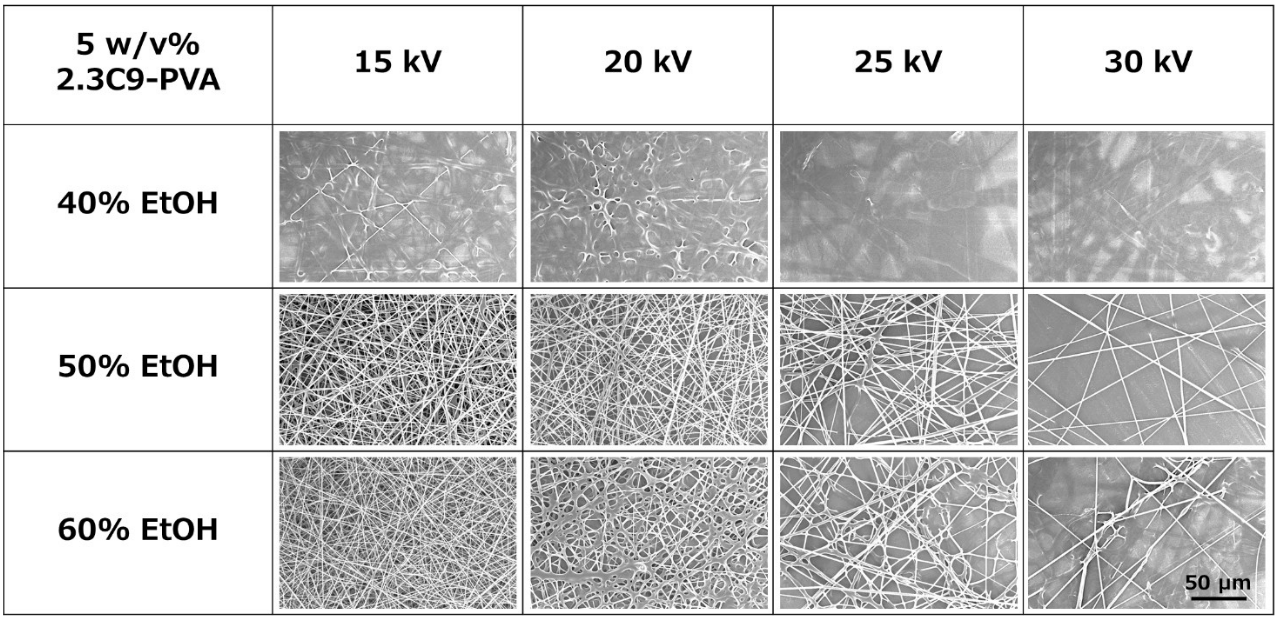

Figure S2. SEM images of electrospun fiber sheets using different 2.3C9-PVA solutions (5 w/v %, 40–60 % EtOH) under the voltage of 15–30 kV (scale bar = 50 μm).

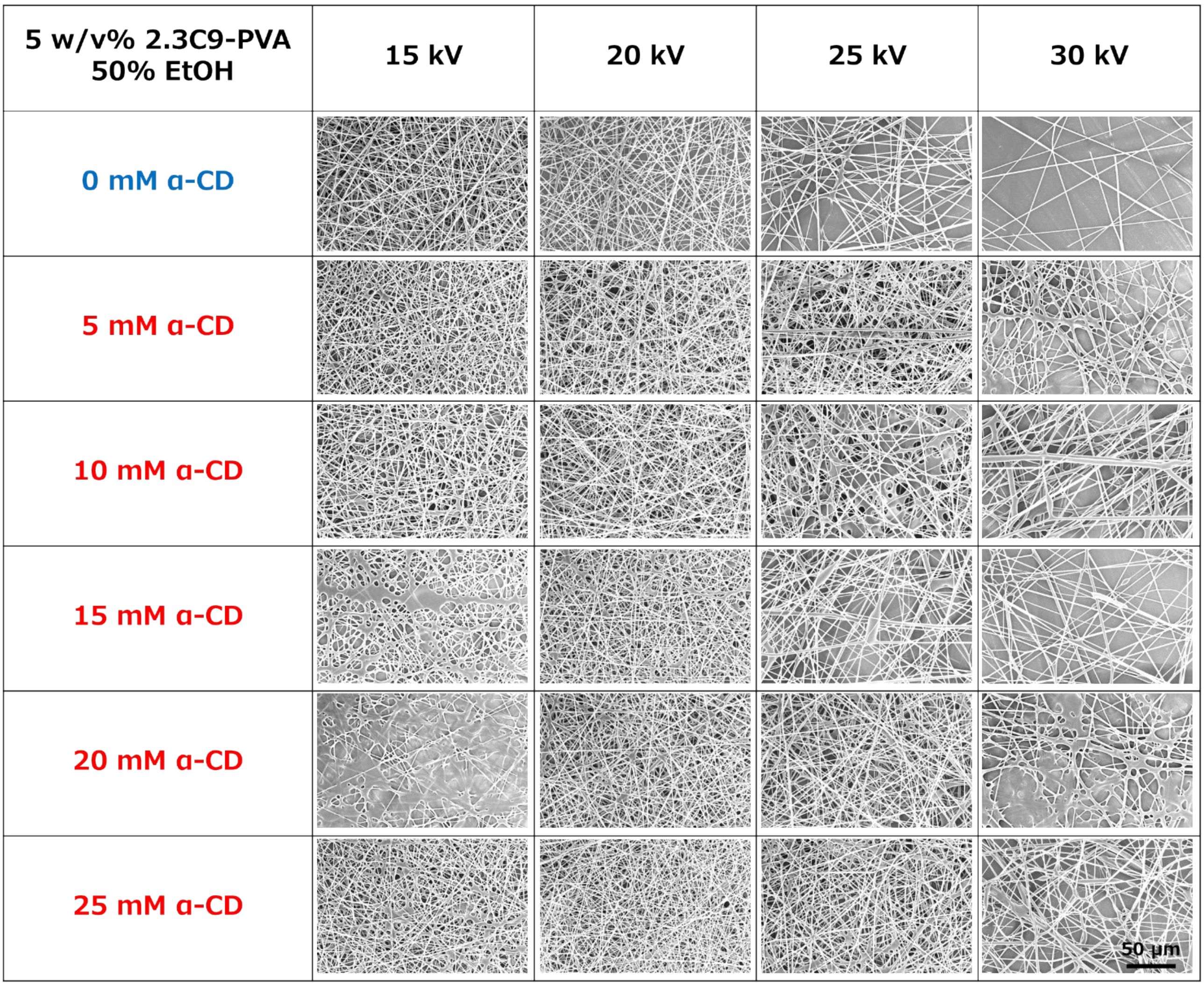

Figure S3. SEM images of electrospun fiber sheets using different α-CD/2.3C9-PVA solutions (5 w/v % 2.3C9-PVA, 50 % EtOH, 0–25 mM α-CD) under the voltage of 15–30 kV (scale bar = 50 μm).
